# Salivary Histamine Levels in Patients with Oral Lichen Planus Lesions

**DOI:** 10.3390/medicina60071038

**Published:** 2024-06-25

**Authors:** Małgorzata Radwan-Oczko, Anna Rybińska, Agata Mierzwicka, Irena Duś-Ilnicka

**Affiliations:** 1 Department of Oral Pathology, Wrocław Medical University, ul. Krakowska 26, 50-425 Wrocław, Poland; malgorzata.radwan-oczko@umw.edu.pl (M.R.-O.); anna.szyczygielska@umed.wroc.pl (A.R.); 2 Department of Endocrinology, Diabetology and Isotope Treatment, Wybrzeże L. Pasteura 4, 50-367 Wrocław, Poland; agata_oli@wp.pl

**Keywords:** histamine, lichen planus, oral, saliva, autoimmune disease, salivary proteins

## Abstract

*Background and Objectives:* An oral lichen planus (OLP) chronic lesion refers to a group of oral potentially malignant disorders (OPMDs) that still lack a proper understanding from the point of view of relevant biomarkers for diagnostics and prognosis. The aim of the study was to assess the salivary histamine levels in patients with oral lichen planus lesions. *Materials and Methods:* The study included a group of 76 patients with oral lichen planus. General diseases and medication taken, smoking habits, severity of pain assessed using a visual analogue scale (VAS), oral hygiene status, and duration of OLP were evaluated. ELISA diagnostics for histamines in saliva levels were assessed. *Results:* The histamine levels in the OLP group were higher (0.468) in comparison with the control group (0.056), without a statistically significant value *p* = 0.090 (Mann–Whitney U Test). The median age of 76 OLP patients was 63 years (min 22.0–max. 81), with the biological sex being 80.3% females and 15 19.7% males. The average duration of OLP lesion presence was 29.4 months (SD 37.1) and the median value was 14.5 months. The median of the VAS was 3.0. OLP assessment in accordance with the Malhotra methodology showed the highest frequency—30.3% for only two of the point areas involved and 17.1% for three points. Clinical assessment of the different OLP grades, severity, and oral site involvement and the VAS in correlation with histamine salivary levels showed a lack of statistical significance in the investigated population. *Conclusions:* Undertaking further research could provide further possibilities for searching for general factors in OLP development.

## 1. Introduction

Histamine is a biogenic amine (also known as 2-[4-imidazolyl]-ethylamine) that was first synthesized in the early 1900s [1] when Dale and Laidlaw discovered a substance that was capable of inducing smooth muscle contraction during the preparation of isolated guinea pig ileum in 1910 [2]. Since then, its functions have started to be discovered and better described. Histamine plays a central role in various physiological and pathophysiological processes, especially acting as a mediator in allergic diseases and inflammation [3]. Histamine, released from mast cells, leads to bronchoconstriction, vasodilation, the “triple response” (erythema, flares, and wheals), and pain via irritation of nociceptive nerve fibers [4]. Recent research has also indicated its importance in angiogenesis in tumor models and wound healing, among others [1,5]. Histamine exerts its effects by binding to its four receptors (H1R, H2R, H3R, and H4R) on target cells in various tissues. These receptors differ in their pharmacology and signal transduction properties. As a result of the varying expression of receptors, the effects of histamine may be mixed or even have the opposite effect (Table 1).

In the past, research has mainly focused on the expression of H1R and H2R; however, the characterization of H4R has now shed new light on the importance of histamine for the functioning of immune cells [3]. The H4 receptor is preferentially expressed in various cells of the immune system and mast cells and induces the chemotaxis of, for example, mast cells and eosinophils [3]. It has also been identified in lymphocyte T cells, dendritic cells, and basophils [6].

**Table 1:** Histamine receptors and their signal transduction and physiological function in the human body [3,7,8].

Receptor	Expression Tissue or Organ	Intracellular Signal Cascade	Physiological Function
H1R	Smooth muscle in the respiratory, gastrointestinal, and urogenital tracts; endothelial cells, epithelial cells, nerve cells, neutrophils, eosinophils, monocytes, macrophages, dendritic cells, and T and B cells	Ca^2+^↑, phospholipase D, phospholipase A_2_, NFκB	Contraction of smooth muscle Increase in capillary permeability Classic symptoms of allergic reactions (rash, edema, pruritus, and pain) Sleep–wake cycle
H2R	Smooth muscle in the respiratory tract and vessels; parietal cells in gastric mucosa, hepatocytes, chondrocytes, endothelial cells, epithelial cells, nerve cells, neutrophils, eosinophils, monocytes, macrophages, dendritic cells, and T and B cells	cAMP↑, adenylate cyclase, c-Fos, c-Jun, PKC, p70S6K	Relaxation of smooth muscle Glandular secretion (gastric acid secretion) Chronotropic and inotropic heart effects
H3R	Histaminergic neurons, eosinophils, monocytes, dendritic cells	cAMP↓, Ca^2+^↑, MAP kinase	Release of neurotransmitters Regulation of cognitive function Regulation of sleep Regulation of food intake
H4R	Bone marrow, endocrine cells in the gastrointestinal tract (intestinal and spleen), mast cells, eosinophilic basophils, dendritic cells, T lymphocytes, monocytes, neutrophils, nerve cells, and dermal fibroblasts	cAMP↓, Ca^2+^↑, AP-1↑	Regulation of the immune system by the production of cytokines and chemokines Possible regulation of haematopoiesis Regulation of neuropathic pain

**Figure 1:**
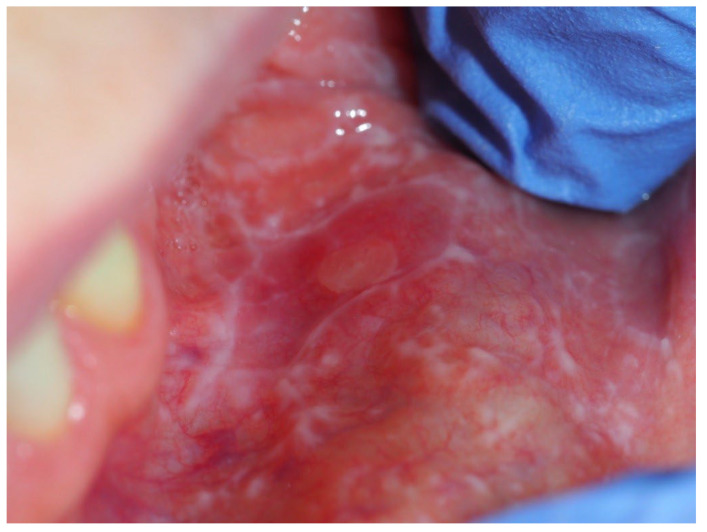
Clinical representation of oral lichen planus in patients of the Oral Pathology Department.

Oral lichen planus (OLP) is sufficiently clinically described as chronic oral mucosa disease classified as oral potentially malignant disorder (OPMD), with a prevalence of 1–2% of the adult population [9,10]. It has been assessed as an autoimmune disease characterized by Salem et al. as a band-like T-cell infiltrate shown below the apoptotic epithelial cells and degenerated basement membrane of the lesion [11]. OLP is present in different forms (from white reticular, plaque lesions or red–white atrophic, erosive, and the most advanced bullous lesions; one of the representative results is shown in Figure 1). All sites of oral mucosa can be involved, but the buccal mucosa, lateral borders of the tongue, dorsal tongue, or gingiva are the typical localizations [12]. Although many findings from clinical trials, case reports, observational studies, and other investigations of different markers and diagnostic methods have been presented in the literature [13,14], where authors highlight the many possible elements that seem to be considered risk factors in the development and course of this pathology, exact knowledge about OLP is still not complete. Because of this, treatment is mainly symptomatic without knowledge of when and why OLP lesions become more advanced [15,16,17].

The role of histamine has been investigated in connection with oral pathologies [18]. Already the first papers published in 1978 and 1984 concerned periodontal diseases [19]. The authors found that patients with gingivitis and periodontitis had higher salivary histamine levels, which provided an important indicator that histamine influences inflammatory response [16]. Then, the study by Minami et al. showed that human gingival fibroblasts express the H1 receptor and that histamine induces interleukin—IL-8 production through the H1 receptor [17]. There are also authors who have pointed out that salivary histamine may be used as a predictive factor of periodontal disease [16]. Another study showed in the salivary glands of patients with Sjögren’s syndrome the presence of incomplete histamine-producing cells [4]. The study by Medina et al. pointed out that histamine completely reversed radiation-induced decreased salivary secretion [20]. Similarly, Prestifilippo et al. described methacholine-induced salivary reduction reversed by histamine, which was associated with an increased proliferation rate in submandibular glands [20]. A newer report by authors in Finland indicated that oral epithelial cells are also incomplete histamine-producing cells [18].

Salem et al. found that healthy oral epithelial cells are equipped with H4 receptors, but in OLP lesions, an increased number of activated mast cells (MCs) is associated with increasing loss of the epithelial H4 receptor [11].

In the continuation of the next study, Salem et al. [11] concluded that human oral keratinocytes (HOKs) are histamine-producing cells, express high-affinity histamine H4 receptors, and are equipped with it. The number of these receptors is decreased and their expression and function are abnormal in patients with OLP. What is more, histamine N-methyltransferase (NHMT)—the enzyme that metabolizes endogenic histamine—is also decreased in OLP [21]. In this way, the endogenous histamine level may be increased, which could influence epithelial integrity and lead to disease development [21].

OLP is the oral pathology of the immunological and inflammatory reaction. The role of histamine in connection with pathologies is not clear. The aim of the study was to assess the salivary histamine levels in patients with oral lichen planus lesions.

### Aim of the Study

OLP is the oral pathology with the immune mediated pathogenesis [22,23,24]. The role of histamine in connection with pathologies is not clear. The aim of the study was to assessed the salivary histamine level in patients with oral lichen planus lesions.

## 2. Materials and Methods

### 2.1. Patients and Control Group Description

The study was designed as a case–control study. In the investigated group, 76 patients with oral lichen planus who attended an oral pathology outpatient clinic in the years 2021–2022 were recruited. The subjective examination included: age, gender, information about general diseases and medication taken, and smoking habits. The control group consisted of 20 patients—generally healthy. In both groups, the exclusion criteria were as follows: pregnancy or breastfeeding, ongoing general use of antibiotics, active inflammation of the respiratory system, pollen or other allergies, or taking antiallergic medication—on the basis of the patients’ information—and patients with OLP lesions in direct contact with amalgam fillings or metal restorative materials. Patients in both groups were assessed under the same conditions.

### 2.2. Clinical Examination and Bioethical Agreement

Evaluation of the activity of the disease, on the basis of the adopted scoring methodology of Malhotra et al. [25,26], was calculated with the area involved (maximum of 12 points), grades, and the severity of the disease. Furthermore, the type of oral complaint, such as xerostomia feelings, burning sensation presence, and others, and the severity of pain on the basis of the visual analogue scale (VAS), self-assessment of oral hygiene, and duration of the presence of OLP were also considered [27]. The clinical and histopathological diagnoses were made based on modified World Health Organization diagnostic criteria for OLP [28]. Clinical investigation was performed by a specialist in oral mucosal pathologies. OLP diagnoses were made when typical bilateral and symmetrical reticular, popular, plaque-like erythematous or ulcerative lesions were present. The histopathological investigation was performed by a specialist in pathomorphology, with the description of the findings characteristic of this pathology.

The assessment of the sites involved was recorded. The study was conducted according to the principles of the Declaration of Helsinki. Approval of the Ethics Committee of the Medical University to perform this study was obtained (decision number: KB 1022/2021). Written informed consent was provided by all patients and healthy controls. Approval of the patients and consent for publication were provided before the analysis of the collection of the biomaterial.

### 2.3. Saliva Collection Protocol

Patients were recruited from the Oral Pathology Department within the visit range of the entire workday. The decision was made, since OLP is a rare OPMD, not to exclude any patients because of circadian rhythm differences between the patients’ dates of coming to the department for the dental visit. Patients included in this study attended the Oral Pathology Department in the time range from 9 a.m. to 3 p.m. Before saliva collection, personnel assured that the patients had withheld from eating and drinking for at least two hours. The same protocol referred to brushing their teeth and using any mouthwash prior to the visit to the dental studio according to a previously established protocol [29]. After 5 min of rest following clinical examination, the patients were asked to rinse their mouth with lukewarm water and to then spit out approximately 2–5 mL of unstimulated saliva into an Eppendorf-like tube in a sitting position and leaning slightly forward. An identification number was assigned to each patient to ensure a pseudo-anonymized process. After collection, the saliva samples were immediately centrifuged at 10,000× *g* for 10 min at 4 °C. The supernatant was distributed into two sterile aliquot tubes of 1.5 mL and conserved at −80 °C until use.

### 2.4. Histamine Saliva Level Diagnostics

In this study for the quantitative determination of human histamine concentrations in saliva samples, we used a commercially available Human Histamine (HIS) kit (catalogue number: E01H0013) (BlueGene For Life Science (Shanghai, China)) based on solid-phase enzyme-linked immunosorbent assay (ELISA). The histamine ELISA kit applies the competitive enzyme immunoassay technique by utilizing an anti-HIS antibody and an HIS-HRP conjugate. The determinations were carried out in accordance with the manufacturer’s instructions. In the first step, the standards and test samples were incubated at 37 °C together with HRP-labeled histamine on pre-coated plates. After the incubation period, the wells were decanted and washed. Then, the wells were incubated with a substrate for the HRP enzyme. The product of the enzyme–substrate reaction formed a blue-colored complex. Finally, a stop solution was added to stop the reaction, which turned the solution color to yellow. The optical density (O.D.) was measured spectrophotometrically at 450 nm using a The Agilent BioTek Epoch microplate spectrophotometer (EPOCH). The intensity of the color was inversely proportional to the histamine concentration because histamine from samples and HRP-labeled histamine compete for the anti-HIS antibody binding site. A histamine log-linear regression standard curve was generated by plotting the optical density (450 nm) obtained for each of the six standard concentrations on the vertical (Y) axis versus the corresponding concentration on the horizontal (X) axis (25, 10, 5, 2, 1, and 0 ng/mL) using statistical software Statistica 13.1 and 13.3. The assay range was 0–25 ng/mL with the sensitivity of 0.1 ng/mL. The tests were performed under controlled conditions, the coefficient of determination of the standard curve was ≥0.95, and the highest O.D. was more than 1.

### 2.5. Statistical Methods

For each parameter, the mean (X), median (M), standard deviation (SD, range (min and max)), and lower and upper quartiles (25Q and 75Q) were calculated.

The normality of distribution was tested with the Shapiro–Wilk test. The homogeneity of variance was determined using Levene’s test.

Statistical significance between medians for different groups was calculated using the non-parametric Mann–Whitney U test. The relationship between two parameters was assessed using correlation analysis, and Spearman correlation coefficients were calculated. A *p*-value of less than 0.05 was required to reject the null hypothesis. Statistical analysis was performed using the Statistica Ver. 13.3. software package.

## 3. Results

### 3.1. Description of the Study and Control Group Results

In the study, the median age of 76 OLP patients was 63 years (min 22.0–max 81.0), and in the control group of 20 subjects, 33 years (min. 22.0–max 77.0) (*p* = 0.0000 (Mann–Whitney U test). In the study group, there were 61 (80.3%) females and 15 (19.7%) males and 15 (75%) and 5 (25%) in the control group, accordingly. The age difference between the OLP individuals and control group individuals was an effect of recruitment based on the following criteria for the control group: (1) all individuals presenting with oral mucosa changes were excluded from the research and (2) all individuals taking medication related to their general disease, and as such possibly influencing their histamine levels through general medication, were excluded from the study.

The average duration of OLP lesion presence was 29.4 months (SD 37.1) and the median value was 14.5 months. The median value of the pain score on the VAS was low, only 3.0. The histamine level in the OLP group was higher, 0.468 (0.000 ÷ 0.885), in comparison with the control group, 0.056 (0.000 ÷ 0.427), and only approached a statistically significant value, *p* = 0.090 (Mann–Whitney U test).

General disease and medication were declared by 68.4% of individuals in the investigated group. The median age of patients taking general medication was higher in comparison with individuals without general diseases—65.0 vs. 60.0, accordingly. Only 6.6% of individuals were smokers and all of them were men. During self-assessment, 47.4% of patients reported very good oral hygiene, the other 42.1% reported a satisfactory oral hygiene state, and only 10.5% described their oral hygiene as unsatisfactory. In the control group, nobody was a smoker, and 60.8% of subjects reported very good oral hygiene and the other subjects indicated a satisfactory oral hygiene state.

### 3.2. Severity of the Clinical Outcome of the Disease

Local symptoms of the disorder and discomfort present in the oral cavity were reported by 84.2% of patients. The most common feeling was a burning sensation felt by 39.5%; however, a dry mouth feeling was reported by only 9.2% of patients. OLP lesions mainly involved several different oral mucosa sites in 60.6% of cases. The presence of lesions only in the buccal mucosa was seen in 35.5%, and the tongue was the sole site involved in 3.9% of patients.

OLP assessment in accordance with the Malhotra [25] methodology showed the highest frequency—30.3% for only 2 points for the area involved and the next 17.1% for 3 points with 12 as the maximum. These values of the area points involved indicate the presence of 1–3 lowest grading in the highest frequency in 57.9% of cases, middle 4–6 grading in 35.5% of patients, and the highest 7–12 grading in only 5.3% of patients. Finally, the evaluated severity of OLP lesions was the most commonly present in moderate form in 48.7% of patients, with the mild form noted in 35.5%; thus, a lower number of cases and the severe form were present in only 15.8% of cases (see Table 2 and Table 3).

The results of the relationship between histamine levels and the investigated parameters are shown in Table 4. There was a statistically negative correlation only between histamine levels and the patient’s age. There were also negative correlations observed but without statistical importance between the evaluated salivary histamine levels and gender, the duration of disease, the presence of general disease, level on the VAS, OLP severity, and oral mucosa site involvement without statistical importance.

## 4. Discussion

To the best of our knowledge, no previous study has focused on examining salivary histamine levels as a factor in OLP course. The analysis of salivary histamine levels in reference to the clinical form and activity of the OLP lesions and to the VAS showed no statistical correlation. Despite the lack of statistical significance between histamine levels and the investigated parameters, this work presents the direction for further investigations of salivary histamine levels in other different oral cavity pathologies as well. In the present work, we found higher histamine levels in patients with OLP lesions in comparison to the control group, but this difference was not statistically significant. Based on the observations and outcomes of their studies, Salem et al. [11,21] showed a decrease in the OLP lesion’s histamine H4 receptor, which is involved in the maintenance of healthy oral mucosa, and concluded that a high histamine concentration downregulates epithelial adhesion molecules. Additionally, in this mucosal pathology, HOKs might exhibit an increased level of endogenous histamine and contribute to inducing oral lichen lesions. Regarding these descriptions, we found that the assessment of salivary histamine levels in OLP patients is interesting. However, the determination of the relationship between higher histamine levels and the investigated parameters failed in the present study. As shown by the Spearman’s correlation coefficient of the age of the patients in the current results presented herein, the salivary histamine levels were higher in older patients with OLP, but the older the patient with OLP, the lower the salivary histamine levels in the higher quartile of the results. The probable causes of those changes are yet to be understood, and a bigger research group should be considered to establish the reason for this.

In our study, the median age in OLP patients was 63 years (min 22.0–max 81.0), and consisted of 80.3% women, which is in concordance with Park et al. in reference to the division of biological sex (69.9% of women) but with slight age differences among the groups (57.6 years of age in the work by Park et al.) [30]. Our research was a cross-sectional study that included participants visiting the Oral Pathology Department in the three mln regions of Poland. Our finding of a higher number of perimenopausal and menopausal patients with OLP is in concordance with the study by Mohan et al., who also provided results about the higher age of OLP patients than in the general population [31]. The authors also stated that the incidence of OLP can be mediated by decreased levels of estrogen and progesterone in this group of individuals [31]. Those parameters (age and menopausal status) might not only be related to the initial diagnostic process but, as described by Park et al., may also be relevant as the treatment outcomes of OLP were significantly influenced [30].

In the present research, in the majority of cases—60.6% of the investigated group—lesions were present in different sites of the oral cavity. However, in the majority of patients, moderate or mild OLP severity was found, and the severe form was present only in 15.8% of patients. Also, the value of the median of pain with the use of VAS provided by patients was low. On the other hand, as many as 84.2% of patients described different local symptoms in the oral cavity, with the majority reporting a burning sensation. Xerostomia was observed and reported by 9.2% of patients in our study; no patient from the control group declared such an observation. Similar findings were presented by Larsen et al. but in a higher percentage [32]. Xerostomia can be related to general medication and general disease, among others, as highlighted in a recent manuscript describing oral health-related quality of life and xerostomia in type 2 diabetic patients [33]. The authors confirmed that wearing dentures, age, the duration of disease, and the medical management of diabetes mellitus also correlated significantly with oral health-related quality of life questionnaire answers provided by patients for this purpose.

Observations from this study confirm that the evaluated OLP stages were not advanced and, what is more, probably without an increased amount of activated mast cells (MCs), which may not have been connected with higher levels of histamine. Histamine levels were significantly lower in connection with older age. It is worth underlining that as many as 68.4% of patients had general diseases and were under different general treatment, i.e., antihypertensive, cardiac medication, and non-steroid anti-inflammatory drugs. These medications in susceptible patients can be causal factors for the development of oral lichenoid drug lesions/reactions (OLL or OLR) [34], which are similar to OLP lesions both clinically and histopathologically [35]. In the investigated group, patients with general disease were older although not significantly. It might be presumed that in some of these patients, OLL lesions rather than idiopathic oral lichen planus lesions could be present. And from this point of view, these patients might have lower than normal histamine levels not correlated with OLP disease.

Based on the observations and outcomes of their studies, Salem et al. [11,21] showed a decrease in the OLP lesions’ histamine H4 receptor, which is involved in the maintenance of healthy oral mucosa, and concluded that a high histamine concentration downregulates epithelial adhesion molecules. Additionally, in this mucosal pathology, HOKs might exhibit an increased level of endogenous histamine and contribute to inducing oral lichen lesions. Regarding these descriptions, we found that the assessment of salivary histamine levels in OLP patients is interesting. However, the determination of the relationship between higher histamine levels with the investigated parameters failed in the present study.

The detection range of the kit used for the analysis of histamine in this research was selected based on a previous attempt to measure histamine concentration in saliva presented in the publication by A. Kejr et al., which indicated that the values may be in the range of 0.31–12.4 ng/mL [36]. As previous research has pointed out, salivary levels may be influenced by smoking in patients with periodontal disease [19,37], and only 6.6% of patients included in our research were smokers. Finally, it is also significant to underline that the most important cellular sources of histamine are mast cells and basophils. Histamine is also released as a result of the action of a variety of physical factors such as extreme temperatures, trauma, and vibration. Some parts of histamine enter the body with food. The most popular histamine-rich products are fish seafood, matured or fermented food (for example cheese, alcohol, and pickles), and some vegetables (for example spinach, tomato, and eggplant) [1]. The production of histamine by bacteria in the human gut is also an important source of histamine and has been shown to influence the immune response [38,39]. All of these agents can have an influence on salivary histamine levels. As discussed in previous research, the number of H4 receptors is decreased and their expression and function are abnormal in patients with OLP. Endogenous histamine levels have been suggested to be increased in such patients, which could influence epithelial integrity, leading to disease development, but this was excluded in the present research.

Recent research has focused on novel markers for analysis of salivary parameters for a better understanding of OPMDs, sometimes also called precursor lesions [40], in view of the risk of transformation to oral squamous cell carcinoma [41,42]. Different OPMDs might have different grades of transformation risk ranging from 0.13% to 17.5%, over time periods of <1 year to >10 years [40]. From a clinical point of view, patients might be aware of the presence of white or white–red patches that persist asymptomatically through the years and can be followed without treatment or treated based on malignancy risk [40,43]. In cases of the positive histopathological assessment of the risk of OPMDs, different treatment approaches have been provided (surgical or pharmacologic treatment) [44,45,46]. Continued monitoring of the pathologically changed oral mucosa, with the analysis of relevant novel markers [47], to measure progression to OSCC at an early stage is important in OPMD management [40]. A special report, the IARC’s Perspective on Oral Cancer Prevention, published recently highlights this neglected area of research, left in between the medical specialties and so strongly related to general health and dentistry [9]. There is a lack of possibility to present a wider discussion about salivary histamine levels in OLP pathology because of the limited number of available studies.

## 5. Conclusions

Although the assessed salivary histamine levels in the investigated group were higher when compared to the control group, this difference was not statistically significant. However, this study brings some novelty. It describes for the first time histamine level assessment in the saliva of OLP patients. The outcomes of this pilot study might be also considered as the proposal for the alternating direction of searching for the unknown general risk factor for oral lichen planus development. Also, the larger sample size of the investigated group could be considered suitable to achieve more dependable outcomes.

## 6. Limitations of the Study

As the authors present this hospital-based study, it comes with the risk of bias in the individuals recruited for the control group. After the exclusion of the individuals recruited from the Oral Pathology Department who underwent the general medication process and presented any changes in the oral cavity, the age difference between the investigated and control groups was bigger. This is not a surprise, as similar findings were presented by Wąsacz et al. where major concerns of patients visiting dental hospitals/dental clinics referred to treatments of pathological causes (periodontal, oral pathologies, prosthetics, or follow-ups) [48,49]. Because of the median age of the patients included herein, further collaboration with the Geriatrics Department to be able to include the individuals in the control group who are unmedicated, without the oral pathologies but with good oral health status, is required for future research projects on OLP. Also, a larger sample size for the investigated group could be considered suitable to achieve more dependable outcomes.

## Figures and Tables

**Table 2:** The values of age, histamine level, VAS, and OLP duration in the investigated groups.

Study Group *n* = 76	Median	Min	Max	25Q	75Q
Age (years) *	63.0	22.0	81.0	54.5	67.5
Histamine level **	0.468	0.00	1.805	0.00	0.885
OLP duration (months)	14.5	1.0	180.0	6.0	36.0
VAS	3.0	0.0	10.00	0.00	5.00
Control group *n* = 20					
Age (years)	33.0	22.0	77.0	24.5	42.5
Histamine level	0.056	0.00	1.642	0.00	0.427
Age of patients with general diseases n = 52	65.0	12.3	81.0	55.0	68.5
Age of patients without general diseases n = 24	60.0	9.6	77.0	53.5	65.0

* *p* = 0.0000; ** *p* = 0.090 (Mann–Whitney U test).

**Table 3:** Parameters of the presence of subjective parameters in the examination of OLP patients and the control group.

Investigated Parameters	OLP	Control
General disease and medication use	68.4%	0.00%
Smoking	6.6%	0.00%
Oral hygiene self-assessment		
Unsatisfactory	10.5%	0.0%
Satisfactory	42.1%	39.2%
Very good	47.4%	60.8%
Local symptom presence	84.2%	NP
Xerostomia	9.2%	NP
Burning sensations	39.5%	NP
Both symptoms	18.4%	NP
Others	17.1%	NP
Localization		
Buccal mucosa	35.5%	NP
Tongue	3.9%.	NP
Different sites involved	60.6%	NP
OLP severity		
Mild	35.5%	NP
Moderate	48.7%	NP
Severe	15.8%	NP

Not present—NP.

**Table 4:** Correlation between salivary histamine levels and the investigated parameters.

		N	R	*p*
Histamines	Gender	76	–0.03	0.823
	Age		–0.24	0.0336
	Disease duration		–0.11	0.324
	General diseases		–0.11	0.334
	Local problems		0.06	0.619
	VAS		–0.07	0.544
	Oral hygiene		–0.05	0.661
	Grading		0.04	0.732
	OLP severity		–0.15	0.181
	Points of the area involved		0.06	0.602
	Site involved/localization		–0.11	0.336

Spearman correlation.

## Data Availability

The datasets supporting the conclusions of this article are included within the article and its additional files.
